# Assessment of Medication Safety Using Only Dispensing Data

**DOI:** 10.1007/s40471-018-0176-6

**Published:** 2018-09-28

**Authors:** Nicole Pratt, Elizabeth Roughead

**Affiliations:** Quality Use of Medicines and Pharmacy Research Centre, School of Pharmacy and Medical Science, University of South Australia, Adelaide, Australia

**Keywords:** Medication safety, Dispensing data, Self-controlled designs, Medication proxy

## Abstract

**Purpose of Review:**

The purpose of this review is to provide an overview of the published studies that have been used to generate evidence on the safety of medicine use when only medication dispensing data are available.

**Recent Findings:**

Medication dispensing databases are increasingly available for research on large populations, particularly in countries that provide universal coverage for medicines. These data are often used for drug utilisation studies to identify inappropriate medicine use at the population level that may be associated with known safety issues. Lack of coded diagnoses, to identify outcomes, and lack of data on confounders can limit use of these data in practice for medication safety assessment. To overcome these issues, studies have exploited the fact that symptoms of adverse effects of medications can be treated with other medications, for example antidepressants to treat depression or oxybutynin to treat urinary incontinence. The challenge of unmeasured confounding has been addressed by implementing self-controlled study designs that use within-person comparisons and provide inherent control for confounding. Prescription sequence symmetry analysis (SSA) is a within-person study design that has been demonstrated as a useful tool for safety signal generation in dispensing data.

**Summary:**

Using medicine initiation as a proxy for the development of adverse events can help to generate evidence of the safety of medicines when only medication dispensing data are available. Careful consideration, however, should be given to the sensitivity and specificity of the proxy medicine for the adverse event and potential for time-varying confounding due to trends in medicine utilisation. Data-mining approaches using dispensing data have the potential to improve safety assessments; however, the challenge of unmeasured confounding with these methods remains to be investigated.

## Introduction

Medication-related problems can lead to significant harm and impact on quality of life. In Australia, around 2% to 3% of all hospital admissions can be attributed to medicine-related problems while around 10% of patients visiting a medical practitioner have experienced an adverse medication event in the last 6 months [1]. Efforts to improve medication safety focus on both identification and quantification of previously unrecognised adverse events of medicines and the clinical risk factors for those events, as well as identification of inappropriate medicine use in practice. Knowledge about the safety of medicines and use of medicines in high-risk populations can help to inform interventions to improve the use of medicines.

In pharmacoepidemiology, the goal of medication safety studies is to generate knowledge about the risks associated with medicine use and factors that may modify that risk. Large-scale health care administrative databases are a convenient source of information to generate this knowledge as they contain individual patient-level data on health services claimed for many millions of patients. These data are routinely collected in many countries with universal health systems and by large private health insurers. Medication safety assessments using these data can be made by linking exposure data, i.e. medicine dispensing data, to outcome data, i.e. hospital admission data; however, this is not always possible, practical or timely particularly if hospital services and pharmaceutical services are subsidised by different payers and require external linkages to bring the datasets together. Regardless of the ability to link to other data sources, individual patient-level dispensing datasets are often available in many countries [2]. These data contain information on the medication dispensed, date of supply, quantity supplied and dose. For example, Australia maintains a national dispensing dataset of medicines subsidised under their Pharmaceutical Benefits Scheme with the collection beginning in 1990 and patient linked since 2002 [3]. The Nordic countries all maintain nationwide prescription datasets [4] as does New Zealand [5], Scotland [6], Ireland [7] and the Asian countries, Korea, Taiwan, and Japan [8, 9] among others.

When only patient-level dispensing data are available, there are two key opportunities for medication safety assessment: medicine utilisation studies and medication safety assessments. Medicine utilisation studies have long been used in pharmacoepidemiology to identify issues with medicine use at the population level that may be indicative of known safety issues such as use in contraindicated populations, use outside of indication (off-label) or subsidy restriction, inappropriate treatment sequences, inappropriate dosing or prevalence of interacting medicines. Medicine utilisation studies can help to identify the potential over-use or under-use of medicines, particularly where they can be compared with estimates of disease prevalence or incidence for the population under study. These studies are an effective strategy to enhance medication safety as they can identify opportunity for intervention and promotion of quality use of medicines. For example, Polluzi et al. [10] used dispensing data across 13 different European countries to determine the extent of use of antihistamine medicines that were associated with safety signals identified through spontaneous reports. Where use is considered to be high, this can then prompt risk minimisation activities by regulators and clinicians. In Australia, the Australian Government Department of Veterans’ Affairs (DVA) funds an ongoing health promotion-based program, Veterans’ Medicines Advice and Therapeutic Education Services (Veterans’ MATES), which implements interventions to improve use of medicines in the Veteran community [11]. Veterans’ MATES interventions are developed based on issues identified in drug utilisation reviews of medication dispensing data and practice change after implementation of the intervention is evaluated [12, 13].

While medicine utilisation studies in large-scale medication dispensing datasets are frequent, fewer studies have associated medicine use with adverse outcomes using only these data. A review of studies utilising the Australian PBS data [3] identified 50 studies that used individual level dispensing data. The majority of these studies (27 studies) examined trends in utilisation and impacts of interventions while only 9 correlated medicine use with outcomes. Similarly, a review of the use of claims data in the Nordic countries [4] including 515 studies from Denmark, Finland, Iceland, Norway and Sweden found that medicine utilisation studies accounted for 44% of all studies with the remainder investigating the effectiveness or safety of medicines; however, many of these linked dispensing data to other datasets to identify outcomes. In Scotland, dispensing of medicines publicly funded by the National Health Service is captured in the Prescribing Information System. These data have been used predominantly for drug utilisation studies [6].

There are two key barriers to generating evidence of medication safety when only dispensing data are available. The first if the lack of coded diagnosis data to identify adverse events and the second is the lack of data on potential confounders. To address these issues, medication safety assessments can be made by exploiting the fact that symptoms of adverse events of medicines can be treated with other medicines and self-controlled designs that eliminate the need to numerically control for confounding can be implemented.

The aim of this article is to provide an overview of studies that have investigated issues of medication safety using only medication dispensing datasets. We investigate the outcomes assessed by these studies and the medication ‘proxies’ used to define these outcomes. We also examine the methodologies that have been used to generate evidence of medication safety with a particular focus on adjustment for unmeasured confounding. Lastly, we discuss the potential for ongoing work in this area.

## Medicine Safety Studies in Dispensing Data

One of the barriers to medicine safety assessment in dispensing data is the lack of diagnosis data to identify adverse events. In order to use dispensing data for safety assessment, one can utilise the fact that symptoms of adverse effects of medicines can be treated in primary care and do not always result in hospitalisation. When the symptoms of an adverse event are misinterpreted as the development of a new unrelated condition and treated with another medicine, this is sometimes referred to in the literature as a prescribing cascade [14]. Examples include dry cough associated with angiotensin-converting enzyme (ACE) inhibitors, which may be managed by antitussive medication, and urinary incontinence associated with cholinesterase inhibitor use for dementia, which may be managed with oxybutynin. Prescribing cascades can be utilised in safety assessments of medicines by examining the rate of initiation of ‘proxy’ medicines that can be used to treat adverse event symptoms, after initiation of the index medicine.

In addition to the lack of diagnosis data for medication safety studies in dispensing data, another limitation is the lack of available information on potential confounders. The advent of self-controlled designs that inherently control for confounders that do not vary over time by making within-person comparisons has meant that safety studies can be implemented in dispensing data without requiring information on measured confounders. Self-controlled study designs include the case-crossover [15], the self-controlled case series [16] and sequence symmetry analysis [17]. All of these designs share a similar characteristic, which is that the patient’s risk of experiencing an adverse event after exposure is compared with another time in that patient’s history (or their future) when they were not exposed. This comparison to one’s-self rather than another individual eliminates the need to numerically adjust for patient characteristics that do not vary over time such as gender, disease severity or frailty, provided the follow-up time is short.

## Sequence Symmetry Analysis

Of the class of self-controlled designs, by far, the most frequently used in medication dispensing data has been the sequence symmetry analysis (SSA) [17, 18•]. SSA aims to identify a pattern in medicine use that suggests a medicine indicative of treatment of an adverse event is initiated more often after exposure than prior to exposure. The statistic of interest is the sequence ratio, which has been described as the ratio of the rate of events in exposed individuals compared to a similar unexposed population in the period of time before the exposure [19]. We identified 31 published SSA studies that used initiation of a medicine as an indicator of an adverse event. All published SSA studies and the indicator medicines that were used are summarised in Table 1. Examples include initiation of antidepressants as an indicator of depression [17, 42, 43], initiation of antitussives as an indicator of dry cough [49, 51] and initiation of glaucoma eye drops as an indicator for glaucoma [53]. Initially, the method was used to explore specific hypotheses; however, of the studies published in the last 5 years, nearly half used a hypothesis generating approach either to determine which medicines were associated with a specified adverse event (e.g. urinary incontinence [26], lower urinary tract infection [27], erectile dysfunction [31] and heart failure [33••]) or to determine potential adverse events associated with a specific medicine (e.g. adverse events associated with novel oral anticoagulants (NOACs) [56••, 57••], or cholinesterase inhibitors [55]). In the studies investigating novel oral anticoagulants, the aim of the analysis was to use SSA as a pharmacovigilance tool to generate safety signals with the newly marketed medicines. In addition to bleeding or stroke events associated with NOACs, which have been well studied in the literature, these studies identified potential safety signals including constipation, depression and nausea [56••, 57••].

**Table 1:** Tabular summary of published studies using the prescription sequence symmetry analysis design to investigate issues of medication safety using only dispensing data

Author	Year	Dispensing database	Exposure	Target outcome	‘Proxy’ medicine name (medicine code/s)
Alimentary tract and metabolism/gastrointestinal					
Hallas [20]	1998	Odense Pharmaco-epidemiologic database (OPED) Database; Denmark	Multiple	Dyspepsia	H2receptor blockers (NR) Proton pump inhibitors (NR) Bismuth preparations (NR) Sucralfate (NR)
Bytzer [21]	2000	Odense Pharmaco-epidemiologic database (OPED) Database; Denmark	Multiple	Functional dyspepsia	Cisapride (NR)
				Nausea	Metoclopramide (NR)
Pratt [22]	2013	Multiple Databases across Asia, Australia, the USA and Sweden; Asian Pharmacoepidemiology Network (AsPEN)	Antipsychotics	Hyperglycaemia	Insulin (ATC codes; A10A)
Hachiken [23]	2013	Computerised prescription order entry system was analysed at the National Cerebral and Cardiovascular Center of Japan	Aspirin	GI complications	H2-receptor antagonists (NR) Proton pomp inhibitors (NR)
Fujimoto [24]	2013	NR	Statin	Storage lower urinary tract symptoms (LUTS)	Storage LUTS (NR); solifenacin succinate flavoxate hydrochloride oxybutynin hydrochloride
Takeda [25]	2014	NR	Aspirin	GI complications	H2-receptor antagonists (NR) Proton pump inhibitors (NR)
Kalish Ellett [26]	2014	Australian Government Department of Veterans Affairs Claims Database; Australia	Multiple	Urinary Incontinence	Oxybutynin (ATC codes, G04BD04)
Hashimoto [27]	2015	Platform for Clinical Information Statistical Information (CISA) database; Japan	Multiple	Lower urinary tract symptoms	Urge incontinence (NR): oxybutynin, propiverine, flavoxate Overactive bladder (NR): flavoxate, tolterodine, solifenacin, imidafenacin Abdominal pressure-induced incontinence (NR): Clenbuterol Overflow incontinence (NR): bethanechol, distigmine, neostigmine Dysuria associated with benign prostatic hyperplasia (BPH) (NR): paraprost, cernitin pollen extract, eviprostat, tamsulosin, naftpidil, silodosin, prazosin, urapidil, terazosin Enuresis imipramine (NR): clomipramine Nocturnal enuresis (NR): amitriptyline
Cardiovascular					
Corrao [28]	2006	National Health System reimbursable drugs of the Lombardia Region; Italy	Fluroquinolone antibacterial agents	Arrhythmia	Ofloxacin, ciprofloxacin, enoxacin, norloxacin, lomefloxacin, rufloxacin, levofloxacin (ATC codes; J01MA)
Wahab [29]	2014	Australian Government Department of Veterans Affairs Claims Database; Australia	Rosiglitazone	Heart failure	Frusemide (ATC codes; C03CA01)
Roughead [30•]	2015	Multiple Databases across Australia, Hong Kong, Japan, Korea, Taiwan; AsPEN	Thiazolidinediones	Heart failure/oedema	Frusemide (ATC codes; C03CA01)
Rasmussen [31]	2015	Danish National Prescription Registry; Denmark	Multiple cardiovascular drugs	Erectile dysfunction	5-Phosphodiesterase inhibitor (ATC codes; G04BE)
Takeuchi [32]	2015	Japan Medical Data Centre Co. Ltd.; Japan	Atypical antipsychotics	Hyperlipidaemia	Antihyperlipidaemia medicines (NR); statins, fibrates ezetimibe, niacin, bile acid sequestrants, probucol, phytosterols, dextran sulphate sodium sulphur, polyene phosphatidylcholine, elastase and eicosapentaenoic acid
Wahab [33]	2016	Australian Government Department of Veterans Affairs Claims Database; Australia	Multiple	Heart failure	Frusemide (ATC code; C03CA01)
Systemic hormonal preparations					
Lai [34]	2013	National Health Insurance Research Database; Taiwan	Antiepileptic drugs	Hypothyroidism	Thyroxine (ATC code; H03AA01)
Pratt [35•]	2015	Multiple databases across Australia, Hong Kong, Japan, Korea, Taiwan; AsPEN	Amiodarone	Hyperthyroidism	Thyroxine (ATC code; H03AA01)
Infection					
Pouwels [36]	2013	University of Groningen ‘InterAction Database’ pharmacy prescription database; Netherlands	Angiotensin-converting enzyme (ACE) inhibitors	Urinary tract infection	Antibiotic; nitrofurantoin (ATC codes; J01XE)
Van Boven [37]	2013	University of Groningen ‘InterAction Database’ pharmacy prescription database; Netherlands	Inhaled corticosteroids (beclomethasone, budesonide, fluticasone, ciclesonide and ICS combination inhalers with long-acting beta agonists)	Oral candidiasis	Oral formulations (ATC codes; NR); Nystatin, miconazole, methylrosaniline and amphotericin B.
Pouwels [38]	2016	University of Groningen ‘InterAction Database’ pharmacy prescription database; Netherlands	Statins	Infection	Antibiotic treatment (ATC code; J01)
Roughead [39•]	2016	Multiple databases across Australia, Korea, Canada, Japan, Taiwan; AsPEN	Proton pump inhibitors	*Clostridium difficile*	Vancomycin (ATC code; J01AX01)
Hendriksen [40]	2017	Danish healthcare and prescription registries; Denmark	Inhaled corticosteroids ATC, ICS: R03BA; ICS1LABA: R03AK	Antifungal therapy	Systemic antifungal medicines: ketoconazole (ATC code; J03BA02), fluconazole (ATC code; J02AC01), itraconazole (ATC code; J02AC02) Oral antifungal; nystatin (ATC code; A07AA02) Miconazole (ATC code; A07AC01)
Musculoskeletal					
Silwer [41]	2006	Odense Pharmaco-epidemiologic database (OPED) Database; Denmark	Statins	Muscle pain	NSAIDs (ATC codes; M01A)
Garrison [19]	2012	British Columbia, Canada PharmaNet database; Canada	Multiple	Nocturnal leg cramps	Quinine (codes; NR)
Nervous system					
Hallas [17]	1996	Odense Pharmaco-epidemiologic database (OPED) Database; Denmark	Multiple cardiovascular drugs	Depression	Antidepressants (ATC codes; NR)
Lindberg [42]	1998	Odense Pharmaco-epidemiologic database (OPED) Database; Denmark	Statins	Depression	Antidepressants (ATC code; N06A)
Hersom [43]	2003	Quintiles Informatics Database; USA	Isotretinoin	Depression	Antidepressants (ATC codes; NR): selective serotonin reuptake inhibitors, secondary and tertiary tricyclics Other antidepressants
Takada [44]	2014	Japan Medical Information Research Institute, Inc. (JMIRI); Japan	Statins	Sleep disturbances (insomnia)	Hypnotic drugs (NR): ramelteon, zolpidem tartrate, zopiclone, eszopiclone, triazolam, etizolam, brotizolam, rilmazafone hydrochloride hydrate, lormetazepam, nimetazepam, flunitrazepam, estazolam, nitrazepam, quazepam flurazepam hydrochloride, haloxazolam
Chen [45]	2015	Clinical Practice Research Datalink; UK	Tramadol	Depression	Antidepressants (NR): tricyclic antidepressants
Takada [46]	2016	Japan Medical Information Research Institute; Japan	Benzodiazepines	Dementia	Anti-dementia medicines: donepezil, galantamine rivastigmine, memantine
Park [47]	2018	National Health Insurance Service-National Sample Cohort; Korea	Proton pump inhibitors	Dementia	Anti-dementia medicines: Medicines to treat dementia (ATC codes; N06D) excluding ginkgo folium (ATC codes; N06DX02)
Nausea/dizziness					
Bytzer [21]	2000	Odense Pharmaco-epidemiologic database (OPED) Database; Denmark	Multiple	Nausea	Metoclopramide (ATC codes; NR)
Caughey [48]	2010	Australian Government Department of Veterans Affairs Claims Database; Australia	Multiple	Dizziness	Prochlorperazine (ATC codes; N05AB04)
Respiratory					
Vegter [49]	2010	‘InterAction Database’ pharmacy prescription database (IADB.nl); Netherlands	Angiotensin-converting enzyme (ACE) inhibitors	Dry cough	Antitussive agents (ATC codes; R05D)
Almqvist [50]	2012	Swedish National Board of Health and welfare Prescribed drug Register; Sweden	Antibiotics	Asthma	Asthma medications; at least 2 dispensings (ATC codes; R03CC, R03AC, R03BA, R03AK, R03DC)
Vegter [51]	2013	University of Groningen ‘InterAction Database’ pharmacy prescription database; Netherlands	Angiotensin-converting enzyme (ACE) inhibitors	Dry cough	Antitussives (ATC codes; NR)
Data mining (multiple outcomes)					
Tsiropolous [52]	2009	Odense Pharmaco-epidemiologic database (OPED) Database; Denmark	Antiepileptic	Multiple	ATC codes; 3 and 4 digit level
Roughead [53]	2012	Australian Government Department of Veterans Affairs Claims Database; Australia	Glaucoma eye drops	Multiple	Multiple conditions (ATC codes; NR) Reactive airways disease, Diabetes, Ischaemic heart disease, Chronic heart failure, Depression
Lai [54]	2014	National Health Insurance Research Database (NHIRD); Taiwan	Sulpiride	Multiple	Extrapyramidal symptoms (NR): trihexyphenidyl Hyperglycaemia: hyperglycaemic agents Hyperprolactinemia: prolactine inhibitors Cardiac arrhythmias: class 1B antiarrythmics agents
Venalainen [55]	2017	Pharmaceutical Benefits Scheme data; Australia	Cholinesterase inhibitors	Multiple	Nausea: antiemetics (ATC codes: A04A, N05AB04, A03FA03, A03FA01) Dyspepsia: histamine 2 and antacids (ATC codes: A02BC, A02BA, A02A) Urinary incontinence: oxybutynin (ATC codes: G04BD04) Seizures: anticonvulsants (ATC codes: N03A) Anxiety: anxiolytics (ATC codes: N05B) Insomnia: hypnotics and sedatives (ATC codes: N05C) Depression:antidepressants (ATC codes: N06A)
Hellfritzsch [56••]	2018	Odense Pharmaco-epidemiologic database (OPED) Database; Denmark	Non-vitamin K antagonist oral anticoagulants (NOACs)	Multiple	All ATC codes
Maura [57••]	2018	French National Healthcare databases (Régime Général); France	Direct oral anticoagulants (DOACs)	Multiple	Major depressive disorders: antidepressants (ATC codes; N06A) Antiglaucoma medications (ATC codes; S01EA03, S01EA05, S01EB01, S01EC03, S01EC04, S01ED01, S01ED02, S01ED03, S01ED05, S01ED51, S01EE01, S01EE03, S01EE04) Composite: drugs for acid-related disorders (ATC codes; A02), drugs for functional gastrointestinal disorders and other antiemetics (ATC codes; A03A, A03E, A03F A04AD), bile therapy (ATC codes; A05AX), drugs for constipation (ATC codes; A06A), antidiarrheals, intestinal anti-inflammatory/anti-infective agents (ATC codes; A07) Composite without drugs for acid-related disorders: as above without ATC code A02 Antiemetics drugs: metoclopramide (ATC codes; A03FA01), domperidone (ATC codes; A03FA03), metopimazine (ATC codes; A04AD05) Drugs for constipation: (ATC codes; A06A)
Nishtala [58]	2017	NZ Ministry of Health prescription database; New Zealand	Amiodarone, Lithium Simvastatin Fluticasone Frusemide	Multiple	Hypothyroidism: thyroxine (NA) Hyperthyroidism: carbimazole (NA) Muscle cramps: quinine sulphate (NA) Oral candidiasis: carbimazole (NA) Hypokalaemia: potassium (NA)
Hoang [59••]	2018	Australian Government Department of Veterans Affairs Claims Database; Australia	Multiple	Multiple	ATC codes
Hallas [60••]	2018	Odense Pharmaco-epidemiologic database (OPED) Database; Denmark	Multiple	Multiple	All ATC codes

*NR* not reported

## Self-Controlled Case Series and Case-Crossover

Very few studies were identified in the literature that used either the self-controlled case series (SCCS) or the case-crossover design where the outcome was identified through dispensing data only. A systematic review of applications of either SCCS or case-crossover (CCO) [61] identified no studies that used only dispensing data to define outcomes. One SCCS study included in the review [62] used prescriptions to identify depression associated with discontinuation of long-term use of glucocorticoids; however, hospitalisation data was also used in a composite outcome. Another review of case-crossover methodologies in medication safety and effectiveness studies [63] identified 70 empirical applications of the method, but included only one study that used medicines dispensing data only to define the outcome. This study [64] examined the risk of flare of inflammatory bowel disease, defined as initiation of a corticosteroid, associated with antibiotic use.

## Discussion

In this review, we have identified that medication safety studies using dispensing data only are possible; however, identification of a ‘proxy’ for identification of an adverse event is required as is an appropriate study design that limits the need to adjust for confounding. The advantage of using initiation of medicines as a proxy for adverse events is that safety signals may be able to be identified more rapidly than waiting for spontaneous reports to be lodged. Additionally, dispensing data are often more timely than other types of health claims data. For example, dispensing data in Australia is made available to researchers with a 3-month delay while hospitalisation data can often be delayed by a year or more. Investigating dispensing data for adverse events has the potential to identify less serious events that may not require hospitalisation such as nausea and vomiting. These symptoms are often frequent and can have a detrimental impact on patient’s quality of life, medication adherence and persistence. We found that symmetry analysis, a technique that analyses patterns in treatment initiations to identify safety signals, has been used widely in dispensing data. This technique has been used both as a tool for directed inquiry and for hypothesis generation. Due to its ease of implementation and minimal data requirement, the method has been used as a tool in the development of a rapid post-market surveillance system across the Asia-Pacific region as part of the Asian Pharmacoepidemiology Network (AsPEN) [22, 30•, 35•, 39•]. SSA has been shown to have moderate sensitivity (61%) and high specificity (93%) for detecting known adverse drug reactions [65] and a recent review concluded that it is a promising method for signal detection in administrative health databases [66•].

To enhance the validity of medication safety studies in dispensing data, further research is required to determine the sensitivity and specificity of the medication ‘proxy’ used for identification of adverse events. Table 1 shows that variations exist in the medicines used to define similar outcomes and similar medicines are used to define different outcomes. Since many dispensing systems do not collect reason for prescription, it is often difficult to determine indications for medicines that can be used to treat multiple conditions and this will impact on medication safety assessments. For example, warfarin can be initiated to treat deep vein thrombosis (DVT) or atrial fibrillation. For some medicines, the pattern of medication use post initiation can help to distinguish indication, for example in the case of warfarin, shorter-term treatment may indicate treatment for DVT while longer-term treatment may indicate atrial fibrillation. Safety signals generated by SSA, when using dispensing data only, have been compared to safety signals generated when hospital data are available. For example, a study by Wahab et al. [33] used SSA to examine the association between medicine use and heart failure using both initiation of frusemide as a ‘proxy’ for heart failure and hospital admission for heart failure. Of 397 medicines in which heart failure was not listed in the product information, signals were generated for 12 medicines using heart failure hospitalisation as the outcome and with 9 medicines using frusemide as the ‘proxy’ for outcome. While there were differences in the medicines identified, prostaglandin eye drops were identified in both analyses. A limitation of utilising dispensing data for medicine safety assessment is that there may be no specific pharmacological treatment for some adverse events or the initial medicine may be discontinued when an adverse event has occurred rather than being treated with another medicine. Additionally, adverse events of medicines may be managed by treatments not recorded in claims data such as over-the-counter treatments or treatments managed within a health practitioner or emergency department visit, for example an Epi-pen administered for anaphylaxis.

Future directions for developing the potential for medication safety studies utilising dispensing data only should concentrate on identifying valid and standardised ‘proxies’ for particular adverse events. Comorbidity scores that use algorithms of medication dispensing to identify clinical conditions may be one avenue to help in this pursuit. Pharmacy-based measures of comorbidity that identify particular diseases based on patterns of medication use have been in existence since the 1990s. The first of these was the Chronic Disease Score (CDS) [67], consisting of 17 comorbidity categories where medicines were used to identify the presence or absence of the category. A version for the US veteran population, that could be calculated from routine dispensing claims data and was known as the Rx-Risk-V index, was developed in 2003; it consisted of 45 categories of comorbidity [68]. Since then, an updated Rx-Risk index [69] and other comorbidity scores that utilise dispensing data including the Drug Derived Complexity Index (DDCI) [70] and the Medicines Comorbidity Index [71] have all been validated as predictors of mortality in dispensing data and may be useful for identifying particular conditions as indicators of adverse outcomes. Additionally, these scores have the potential to be used to adjust for confounding in cohort studies utilising only dispensing data.

While self-controlled designs are advantageous when data on potential confounders are not captured, as is the case with dispensing only data, they do have important assumptions that must be met and these can be restrictive to their use in these data. For example, the SCCS assumes that outcome events are rare or independent of each other, which may not be met where medicines are used for chronic conditions. Medicines that can be used intermittently such as antibiotics (indicative of infection) or intermittent pain medications may be more suitable as outcomes for SCCS. The assumption that consecutive events are independent is not applicable for CCO or SSA as only the first outcome is analysed in these studies. Another assumption of self-controlled designs is that the occurrence of the outcome event should not affect the likelihood of subsequent exposure. This assumption is required for both the SCCS and SSA but is not applicable to the CCO as the CCO design only examines time before an outcome event. In dispensing data, there may be many situations in which this assumption will be violated. For example, if the outcome medicine is contraindicated after initiation of another medicine, studies will be biased towards the null while if the outcome medicine is usually initiated sequentially after another medicine, studies will be biased away from the null. The last assumption of self-controlled designs is that the occurrence of the outcome should not censor the observation period. Again, the CCO is robust to this assumption as only time prior to outcome is analysed. For the SCCS and SSA, this assumption is unlikely to be violated except where death is used as the outcome of interest. For the SCCS, a modified analytic technique has been developed to overcome this problem [72]; however, the SSA cannot be performed at all as clearly there will never be a sequence of events in which medication is initiated after death. Lastly, self-controlled designs using medications as a proxy for outcome will suffer from miss-classification bias due to uncertainty around the onset date of an insidious outcome, e.g. depression, diabetes or cancer [61], particularly if the decision to treat with a medicine occurs well after a condition is recognised and diagnosed, e.g. diabetes which may be managed through diet.

One of the biggest threats to the use of self-controlled designs for studying medication safety in dispensing data is the assumption of no time-varying confounding. When analysing medication treatment patterns, time-varying confounding can be present either when specific treatments are more likely to be initiated in a particular order as patients age or due to underlying trends in medicine utilisation. As patients age, certain medicines may increase in likelihood of prescription in a particular sequence. For example, anti-dementia medicines will be more likely to be initiated at an older age and therefore after other preventative medicines such as statins. Exposure time trends due to marketing campaigns, new medicines entering the market or removal of medicines from the market can also affect the likelihood of specific treatment orders and can result in spurious relationships in the absence of a casual association. For example, if a medicine indicative of an adverse event is increasingly used over time at the population level, then it is more likely that it will be initiated after the medicine of interest just by chance. All of the self-controlled designs have techniques that can be employed to control for underlying trends in medicine use overtime. In SSA, a null sequence ratio can be calculated which estimates the sequence ratio that would be expected due to the underlying trends in medicines in the absence of an association [17, 52]. The effects of age or calendar time on exposure trends can be modelled directly in the SCCS design [73]. In case-crossover studies, a case-time-control design [74] or case-case-time-control design [75] can be implemented. In these designs, the odds ratio from the CCO analysis is divided by the odds ratio for exposure in the risk periods in matched ‘controls’, i.e. those that have not experienced the outcome event at the same calendar time as the case. While these techniques are available, a comparison of the self-controlled designs [76•] found that results of CCO and SSA methods were robust to exposure time trends while SCCS had residual bias with long-term time trends in both exposure and outcome events.

Dispensing datasets often contain information related to patient demographics including data such as date of birth, gender and date of death. When these data are available, it is possible to undertake medication safety research using only dispensing data in which the outcome of interest is death. For example, the CCO method has been used to determine the risk of death associated with use of selective cyclooxygenase-2 inhibitors and nonselective nonsteroidal anti-inflammatory drugs after acute myocardial infarction [77] and the SCCS has been used to investigate the association between bupropion for smoking cessation and death [78].

While many of the studies included in this review used the initiation of a medicine as a proxy for the development of an adverse event, other studies have utilised longer-term patterns in dispensing data to indicate safety issues. For example Joshi et al. [79] utilised changes in drug treatment to infer disease progression or treatment failure in cancer. Another study used patterns of accumulation of cardiovascular diseases, indicated by medications used to treat them, to investigate the effectiveness of modifiable disease progression in statin initiators [80].

## The Future of Medication Safety Studies in Dispensing Data: Hypothesis-Free or Purposeful Inquiry?

As has been the trend in many areas, data-mining approaches have been used to interrogate dispensing data. Hallas et al. [60••] published a hypothesis-free approach to signal detection using prescription sequence symmetry analysis in which every medicine was tested against every other medicine. The results of this experiment showed that while many signals were generated, over half represented already known drug reactions, common treatment pathways or simply good clinical practice. This highlights a limitation of hypothesis-free screening which is the generation of a large volume of non-informative associations that require further clinical review. To address this problem, a study by Hoang et al. [59••] used a supervised machine learning approach to predict potential adverse drug reactions in which models were trained on positive and negative control associations and using domain knowledge databases (e.g. Drug Bank and SIDER). Hoang et al. linked each drug in the dispensing data with the Structured Indications from DrugBank via the medicine ATC Code. The indications were then linked to hierarchies in the medical dictionary for regulatory activities (MedDRA). Supervised ADR classifiers were then used to predict whether sequences of medicine use were potential ADRs given the domain knowledge. The gradient boosting classifier was found to have improved performance over SSA, improving sensitivity by 21% compared to SSA without any loss of specificity. While hypothesis-free approaches to safety signal detection that machine learning allows are likely to enhance detection of medication safety issues, the probabilities produced may be more difficult for clinicians and regulators to interpret and do not provide quantification of the extent of the harm. Machine learning techniques may have a place with dispensing data by ranking the probabilities of signals, thus enabling triage for directed enquiries using other methods, such as cohort studies in linked data in which hospitalisation or diagnosis data are available***.***

## Conclusion

Medication safety assessment in dispensing data has the potential to provide timely evidence to complement spontaneous reports particularly as medicines enter the market. To enhance the validity of medication safety studies, research should focus on validating patterns of medication dispensing as indicators of adverse event occurrence. Self-controlled designs are likely to be the most appropriate approach to generating this evidence as they eliminate the need for confounding adjustment; however, their application may be limited due to their strict assumptions and their potential for bias due to time-varying confounding due to trends in medicine utilisation. While machine learning approaches are likely to be of value in the exploration of safety signals in dispensing data, research should investigate the ability of these techniques to control for confounding. Incorporating electronic domain knowledge bases has the potential to help train machine learning algorithms as well as aid in filtering the large volumes of spurious signals likely to be generated by these analyses.

While generation of evidence of previously unknown safety issues with medicine use are of value, medicine utilisation studies in dispensing data should not be overlooked as a powerful tool to identify patterns of use that may be indicative of already known safety concerns. This evidence can be effective in informing strategies to promote more appropriate prescribing so that harms are avoided.

## References

[1] Australian Commission on Safety and Quality in Health Care (2013). Literature review: medication safety in Australia.

[2] Milea D, Azmi S, Reginald P, Verpillat P, Francois C. A review of accessibility of administrative healthcare databases in the Asia-Pacific region. J Mark Access Health Policy. 2015;3. 10.3402/jmahp.v3.28076 PMC4802693 27123180

[3] Pearson SA, Pesa N, Langton JM, Drew A, Faedo M, Robertson J (2015). Studies using Australia’s Pharmaceutical Benefits Scheme data for pharmacoepidemiological research: a systematic review of the published literature (1987-2013). Pharmacoepidemiol Drug Saf.

[4] Wettermark B, Zoega H, Furu K, Korhonen M, Hallas J, Norgaard M (2013). The Nordic prescription databases as a resource for pharmacoepidemiological researcha literature review. Pharmacoepidemiol Drug Saf.

[5] Horsburgh SMM, Norris P, Harrison-Woolrych M, Tordoff J, Becket G, Heerbison P, Parkin L, Reith D (2009). Prescribing and dispensing data sources in New Zealand: their usage and future directions.

[6] Alvarez-Madrazo S, McTaggart S, Nangle C, Nicholson E, Bennie M (2016). Data resource profile: the Scottish National Prescribing Information System (PIS). Int J Epidemiol.

[7] Sinnott SJ, Bennett K, Cahir C (2017). Pharmacoepidemiology resources in Ireland-an introduction to pharmacy claims data. Eur J Clin Pharmacol.

[8] Lai EC, Man KK, Chaiyakunapruk N, Cheng CL, Chien HC, Chui CS (2015). Brief report: databases in the Asia-Pacific region: the potential for a distributed network approach. Epidemiology.

[9] Kimura T, Matsushita Y, Yang YH, Choi NK, Park BJ (2011). Pharmacovigilance systems and databases in Korea, Japan, and Taiwan. Pharmacoepidemiol Drug Saf.

[10] Poluzzi E, Raschi E, Godman B, Koci A, Moretti U, Kalaba M, Wettermark B, Sturkenboom M, de Ponti F (2015). Pro-arrhythmic potential of oral antihistamines (H1): combining adverse event reports with drug utilization data across Europe. PLoS One.

[11] Roughead EE, Kalisch Ellett LM, Ramsay EN, Pratt NL, Barratt JD, LeBlanc VT (2013). Bridging evidence-practice gaps: improving use of medicines in elderly Australian veterans. BMC Health Serv Res.

[12] Pratt NL, Kalisch Ellett LM, Sluggett JK, Gadzhanova SV, Ramsay EN, Kerr M (2017). Use of proton pump inhibitors among older Australians: national quality improvement programmes have led to sustained practice change. Int J Qual Health Care.

[13] Pratt NL, Kalisch Ellett LM, Sluggett JK, Ramsay EN, Kerr M, LeBlanc VT (2015). Commitment questions targeting patients promotes uptake of under-used health services: findings from a national quality improvement program in Australia. Soc Sci Med.

[14] Rochon PA, Gurwitz JH (2017). The prescribing cascade revisited. Lancet.

[15] Maclure M (1991). The case-crossover design: a method for studying transient effects on the risk of acute events. Am J Epidemiol.

[16] Farrington CP (1995). Relative incidence estimation from case series for vaccine safety evaluation. Biometrics.

[17] Hallas J (1996). Evidence of depression provoked by cardiovascular medication: a prescription sequence symmetry analysis. Epidemiology.

[18] Lai EC, Pratt N, Hsieh CY, Lin SJ, Pottegard A, Roughead EE (2017). Sequence symmetry analysis in pharmacovigilance and pharmacoepidemiologic studies. Eur J Epidemiol.

[19] Garrison SR, Dormuth CR, Morrow RL, Carney GA, Khan KM (2012). Nocturnal leg cramps and prescription use that precedes them: a sequence symmetry analysis. Arch Intern Med.

[20] Hallas J, Bytzer P (1998). Screening for drug related dyspepsia: an analysis of prescription symmetry. Eur J Gastroenterol Hepatol.

[21] Bytzer P, Hallas J (2000). Drug-induced symptoms of functional dyspepsia and nausea. A symmetry analysis of one million prescriptions. Aliment Pharmacol Ther.

[22] Pratt N, Andersen M, Bergman U, Choi NK, Gerhard T, Huang C (2013). Multi-country rapid adverse drug event assessment: the Asian Pharmacoepidemiology Network (AsPEN) antipsychotic and acute hyperglycaemia study. Pharmacoepidemiol Drug Saf.

[23] Hachiken H, Murai A, Wada K, Kuwahara T, Hosomi K, Takada M (2013). Difference between the frequencies of antisecretory drug prescriptions in users of buffered vs. enteric-coated low-dose aspirin therapies. Int J Clin Pharmacol Ther.

[24] Fujimoto M, Higuchi T, Hosomi K, Takada M (2014). Association of statin use with storage lower urinary tract symptoms (LUTS): data mining of prescription database. Int J Clin Pharmacol Ther.

[25] Takada M, Fujimoto M, Hosomi K (2014). Difference in risk of gastrointestinal complications between users of enteric-coated and buffered low-dose aspirin. Int J Clin Pharmacol Ther.

[26] Kalisch Ellett LM, Pratt NL, Barratt JD, Rowett D, Roughead EE (2014). Risk of medication-associated initiation of oxybutynin in elderly men and women. J Am Geriatr Soc.

[27] Hashimoto M, Hashimoto K, Ando F, Kimura Y, Nagase K, Arai K (2015). Prescription rate of medications potentially contributing to lower urinary tract symptoms and detection of adverse reactions by prescription sequence symmetry analysis. J Pharm Health Care Sci.

[28] Corrao G, Botteri E, Bagnardi V, Zambon A, Carobbio A, Falcone C, Leoni O (2005). Generating signals of drug-adverse effects from prescription databases and application to the risk of arrhythmia associated with antibacterials. Pharmacoepidemiol Drug Saf.

[29] Wahab IA, Pratt NL, Kalisch LM, Roughead EE (2014). Comparing time to adverse drug reaction signals in a spontaneous reporting database and a claims database: a case study of rofecoxib-induced myocardial infarction and rosiglitazone-induced heart failure signals in Australia. Drug Saf.

[30] Roughead EE, Chan EW, Choi NK, Kimura M, Kimura T, Kubota K (2015). Variation in association between thiazolidinediones and heart failure across ethnic groups: retrospective analysis of Large Healthcare Claims Databases in six countries. Drug Saf.

[31] Rasmussen L, Hallas J, Madsen KG, Pottegard A (2015). Cardiovascular drugs and erectile dysfunction - a symmetry analysis. Br J Clin Pharmacol.

[32] Takeuchi Y, Kajiyama K, Ishiguro C, Uyama Y (2015). Atypical antipsychotics and the risk of hyperlipidemia: a sequence symmetry analysis. Drug Saf.

[33] Wahab IA, Pratt NL, Ellett LK, Roughead EE (2016). Sequence symmetry analysis as a signal detection tool for potential heart failure adverse events in an administrative claims database. Drug Saf.

[34] Lai EC, Yang YH, Lin SJ, Hsieh CY (2013). Use of antiepileptic drugs and risk of hypothyroidism. Pharmacoepidemiol Drug Saf.

[35] • Pratt N, Chan EW, Choi NK, Kimura M, Kimura T, Kubota K, et al. Prescription sequence symmetry analysis: assessing risk, temporality, and consistency for adverse drug reactions across datasets in five countries. Pharmacoepidemiol Drug Saf. 2015;24(8):858–864. **Asian Pharmacoepidemiology Network (AsPEN) study to compare the consistency of the association between amiodarone and thyroid dysfunction across multiple datasets. Identified a consistent association between amiodarone and thyroxine, as a marker of hypothyroidism.** 10.1002/pds.3780 PMC4690514 25907076

[36] Pouwels KB, Visser ST, Bos HJ, Hak E (2013). Angiotensin-converting enzyme inhibitor treatment and the development of urinary tract infections: a prescription sequence symmetry analysis. Drug Saf.

[37] van Boven JF, de Jong-van den Berg LT, Vegter S (2013). Inhaled corticosteroids and the occurrence of oral candidiasis: a prescription sequence symmetry analysis. Drug Saf.

[38] Pouwels KB, Widyakusuma NN, Bos JH, Hak E (2016). Association between statins and infections among patients with diabetes: a cohort and prescription sequence symmetry analysis. Pharmacoepidemiol Drug Saf.

[39] Roughead EE, Chan EW, Choi NK, Griffiths J, Jin XM, Lee J (2016). Proton pump inhibitors and risk of Clostridium difficile infection: a multi-country study using sequence symmetry analysis. Expert Opin Drug Saf.

[40] Henriksen DP, Davidsen JR, Christiansen A, Laursen CB, Damkier P, Hallas J (2017). Inhaled corticosteroids and systemic or topical antifungal therapy: a symmetry analysis. Ann Am Thorac Soc.

[41] Silwer L, Petzold M, Hallas J, Lundborg CS (2006). Statins and nonsteroidal anti-inflammatory drugs-an analysis of prescription symmetry. Pharmacoepidemiol Drug Saf.

[42] Lindberg G, Hallas J (1998). Cholesterol-lowering drugs and antidepressants--a study of prescription symmetry. Pharmacoepidemiol Drug Saf.

[43] Hersom K, Neary MP, Levaux HP, Klaskala W, Strauss JS (2003). Isotretinoin and antidepressant pharmacotherapy: a prescription sequence symmetry analysis. J Am Acad Dermatol.

[44] Takada M, Fujimoto M, Yamazaki K, Takamoto M, Hosomi K (2014). Association of statin use with sleep disturbances: data mining of a spontaneous reporting database and a prescription database. Drug Saf.

[45] Chen T, Chen L, Knaggs RD (2015). Prevalence of antidepressants prescribed to tramadol users in the UK primary care setting - a prescription sequence symmetry analysis. Value Health.

[46] Takada M, Fujimoto M, Hosomi K (2016). Association between benzodiazepine use and dementia: data mining of different medical databases. Int J Med Sci.

[47] Park SK, Baek YH, Pratt N, Kalisch Ellett L, Shin JY (2018). The uncertainty of the association between proton pump inhibitor use and the risk of dementia: prescription sequence symmetry analysis using a Korean healthcare database between 2002 and 2013. Drug Saf.

[48] Caughey GE, Roughead EE, Pratt N, Shakib S, Vitry AI, Gilbert AL (2010). Increased risk of hip fracture in the elderly associated with prochlorperazine: is a prescribing cascade contributing?. Pharmacoepidemiol Drug Saf.

[49] Vegter S (2010). Misdiagnosis and mistreatment of a common side-effect--angiotensin-converting enzyme inhibitor-induced cough. Br J Clin Pharmacol.

[50] Almqvist C, Wettermark B, Hedlin G, Ye W, Lundholm C (2012). Antibiotics and asthma medication in a large register-based cohort study - confounding, cause and effect. Clin Exp Allergy.

[51] Vegter S, de Boer P, van Dijk KW, Visser S, de Jong-van den Berg LT (2013). The effects of antitussive treatment of ACE inhibitor-induced cough on therapy compliance: a prescription sequence symmetry analysis. Drug Saf.

[52] Tsiropoulos I, Andersen M, Hallas J (2009). Adverse events with use of antiepileptic drugs: a prescription and event symmetry analysis. Pharmacoepidemiol Drug Saf.

[53] Roughead EE, Kalisch LM, Pratt NL, Killer G, Barnard A, Gilbert AL (2012). Managing glaucoma in those with co-morbidity: not as easy as it seems. Ophthalmic Epidemiol.

[54] Lai EC, Hsieh CY, Kao Yang YH, Lin SJ (2014). Detecting potential adverse reactions of sulpiride in schizophrenic patients by prescription sequence symmetry analysis. PLoS One.

[55] Venalainen O, Bell JS, Kirkpatrick CM, Nishtala PS, Liew D, Ilomaki J (2017). Adverse drug reactions associated with cholinesterase inhibitors-sequence symmetry analyses using prescription claims data. J Am Med Dir Assoc.

[56] Hellfritzsch M, Rasmussen L, Hallas J, Pottegard A (2018). Using the symmetry analysis design to screen for adverse effects of non-vitamin K antagonist oral anticoagulants. Drug Saf.

[57] •• Maura G, Billionnet C, Coste J, Weill A, Neumann A, Pariente A. Non-bleeding adverse events with the use of direct oral anticoagulants: a sequence symmetry analysis. Drug Saf. 2018. **Similar to the Hellfritzsch study, this study describes the use of symmetry analysis as a tool to complement post-market surveillance of newly marketed medicines. Identifies asssociations between NOACs and gastrointestinal medicines, antiemetic drugs, drugs for consitipation and antidepressants.** 10.1007/s40264-018-0668-9 PMC6061378 29714004

[58] Nishtala PS, Chyou TY (2017). Exploring New Zealand prescription data using sequence symmetry analyses for predicting adverse drug reactions. J Clin Pharm Ther.

[59] Hoang T, Liu J, Roughead E, Pratt N, Li J (2018). Supervised signal detection for adverse drug reactions in medication dispensing data. Comp Methods Prog Biomed.

[60] •• Hallas J, Wang SV, Gagne JJ, Schneeweiss S, Pratt N, Pottegard A. Hypothesis-free screening of large administrative databases for unsuspected drug-outcome associations. Eur J Epidemiol. 2018. **The first study to perform a sequence symmetry analysis for every combination of medicines for every ATC code. Of 186,758 associations tested with 29,891,212 incident drug therapies, 43,575 (23.3%) showed meaningful effect size. 47% of the top 200 drug associations represented unknown associations. Demonstrates usefulness of sequence symmetry analysis as a signal detection tool however will require significant post-hoc review of signals.**

[61] Gault N, Castaneda-Sanabria J, De Rycke Y, Guillo S, Foulon S, Tubach F. Self-controlled designs in pharmacoepidemiology involving electronic healthcare databases: a systematic review. BMC Med Res Methodol 2017;17. 10.1186/s12874-016-0278-0 PMC5299667 28178924

[62] Fardet L, Nazareth I, Whitaker HJ, Petersen I (2013). Severe neuropsychiatric outcomes following discontinuation of long-term glucocorticoid therapy: a cohort study. J Clin Psychiatry.

[63] Consiglio GP, Burden AM, Maclure M, McCarthy L, Cadarette SM (2013). Case-crossover study design in pharmacoepidemiology: systematic review and recommendations. Pharmacoepidemiol Drug Saf.

[64] Aberra FN, Brensinger CM, Bilker WB, Lichtenstein GR, Lewis JD (2005). Antibiotic use and the risk of flare of inflammatory bowel disease. Clin Gastroenterol Hepatol.

[65] Wahab IA, Pratt NL, Wiese MD, Kalisch LM, Roughead EE (2013). The validity of sequence symmetry analysis (SSA) for adverse drug reaction signal detection. Pharmacoepidemiol Drug Saf.

[66] Arnaud M, Begaud B, Thurin N, Moore N, Pariente A, Salvo F (2017). Methods for safety signal detection in healthcare databases: a literature review. Expert Opin Drug Saf.

[67] Von Korff M, Wagner EH, Saunders K (1992). A chronic disease score from automated pharmacy data. J Clin Epidemiol.

[68] Sloan KL, Sales AE, Liu CF, Fishman P, Nichol P, Suzuki NT, Sharp ND (2003). Construction and characteristics of the RxRisk-V: a VA-adapted pharmacy-based case-mix instrument. Med Care.

[69] Pratt NL, Kerr M, Barratt JD, Kemp-Casey A, Kalisch Ellett LM, Ramsay E, Roughead EE (2018). The validity of the Rx-Risk Comorbidity Index using medicines mapped to the Anatomical Therapeutic Chemical (ATC) classification system. BMJ Open.

[70] Robusto F, Lepore V, D’Ettorre A, Lucisano G, De Berardis G, Bisceglia L (2016). The Drug Derived Complexity Index (DDCI) predicts mortality, unplanned hospitalization and hospital readmissions at the population level. PLoS One.

[71] Narayan SW, Nishtala PS (2017). Development and validation of a Medicines Comorbidity Index for older people. Eur J Clin Pharmacol.

[72] Farrington CP, Whitaker HJ, Hocine MN (2009). Case series analysis for censored, perturbed, or curtailed post-event exposures. Biostatistics.

[73] Whitaker HJ, Farrington CP, Spiessens B, Musonda P (2006). Tutorial in biostatistics: the self-controlled case series method. Stat Med.

[74] Suissa S (1995). The case-time-control design. Epidemiology.

[75] Wang S, Linkletter C, Maclure M, Dore D, Mor V, Buka S, Wellenius GA (2011). Future cases as present controls to adjust for exposure trend bias in case-only studies. Epidemiology.

[76] Takeuchi Y, Shinozaki T, Matsuyama Y (2018). A comparison of estimators from self-controlled case series, case-crossover design, and sequence symmetry analysis for pharmacoepidemiological studies. BMC Med Res Methodol.

[77] Gislason GH, Jacobsen S, Rasmussen JN, Rasmussen S, Buch P, Friberg J (2006). Risk of death or reinfarction associated with the use of selective cyclooxygenase-2 inhibitors and nonselective nonsteroidal antiinflammatory drugs after acute myocardial infarction. Circulation.

[78] Hubbard R, Lewis S, West J, Smith C, Godfrey C, Smeeth L (2005). Bupropion and the risk of sudden death: a self-controlled case-series analysis using the Health Improvement Network. Thorax.

[79] Joshi V, Adelstein BA, Schaffer A, Srasuebkul P, Dobbins T, Pearson SA, the Elements of Cancer Care (EoCC) Investigators (2017). Validating a proxy for disease progression in metastatic cancer patients using prescribing and dispensing data. Asia-Pac J Clin Oncol.

[80] Lavikainen P, Korhonen MJ, Huupponen R, Helin-Salmivaara A (2015). Accumulation of cardiovascular and diabetes medication among apparently healthy statin initiators. PLoS One.

